# Impact of Packaging Material on Polyphenol Preservation and Environmental Sustainability in Fresh-Cut Apples

**DOI:** 10.3390/molecules31162845

**Published:** 2026-08-14

**Authors:** Lucia Maddaloni, Giuliana Vinci, Paola Russo, Giuseppina Adiletta, Nicholas Torchia, Sabrina Antonia Prencipe

**Affiliations:** 1 Department of Experimental Medicine, Sapienza University of Rome; Viale Regina Elena 324, 00161 Rome, Italy; 2 Department of Management, Sapienza University of Rome, Via del Castro Laurenziano, 9, 00161 Rome, Italy; giuliana.vinci@uniroma1.it; 3 Department of Chemical Engineering Materials Environment, Sapienza University of Rome, Via Eudossiana 18, 00184 Roma, Italy; paola.russo@uniroma1.it (P.R.); giuseppina.adiletta@uniroma1.it (G.A.); 4 PhenoFarm Srl, Strada Provinciale per Orvinio, 02038 Scandriglia, Italy; ninni.torchia2@gmail.com

**Keywords:** apple fruit, polyphenols, antioxidant activity, food-derived bioactive compounds, shelf life, life cycle assessment, sustainable films

## Abstract

Background: Fresh-cut apples are highly susceptible to quality deterioration due to enzymatic browning and oxidative degradation of bioactive compounds. This study investigated the effects of conventional polyethylene packaging (PE, Pack 1) and two innovative biodegradable packaging materials (Pack 2 and Pack 3) on the stability of bioactive compounds in fresh-cut Golden Delicious apples during refrigerated storage. Individual phenolic compounds ((+)-catechin, caffeic acid, (−)-epicatechin, *p*-coumaric acid, rutin, and quercetin) were quantified by HPLC-PDA, while spectrophotometric assays were used to determine total phenolic content (TPC), total flavonoid content (TFC), and antioxidant capacity (ABTS and DPPH). The environmental performance of the packaging materials was assessed through Life Cycle Assessment (LCA) using SimaPro v.9.5.5. Results: Polyphenol stability was significantly influenced by packaging and storage time (*p* < 0.001). Compared with fresh-cut apples at t0, Pack 2 promoted a 17.4% increase in total phenolic content after 21 days, whereas Pack 1 and Pack 3 showed reductions of 31.0% and 37.6%, respectively. HPLC analysis revealed compound-specific responses, with rutin and quercetin being markedly better preserved in Pack 3 after 21 days (17.65 and 1.93 mg/100 g, respectively) than in Pack 1 (0.67 and 0.19 mg/100 g, respectively). Two-way ANOVA confirmed significant effects of storage time, packaging, and their interaction on TPC, TFC, ABTS activity, and all individual phenolic compounds (*p* < 0.001), whereas DPPH activity was not significantly affected (*p* > 0.05). Pearson correlation (TPC–ABTS, r = 0.6885, *p* < 0.001) and principal component analysis indicated that antioxidant capacity was more closely associated with the qualitative phenolic profile than with total phenolic concentration alone. LCA highlighted environmental trade-offs among the packaging systems: Pack 1 showed lower impacts in several categories, Pack 2 displayed an intermediate environmental profile, whereas Pack 3 reduced dependence on fossil resources but exhibited higher land- and water-use impacts together with limitations related to end-of-life management. Conclusion: Packaging materials significantly affected the preservation of phenolic compounds and antioxidant activity in fresh-cut apples while exhibiting distinct environmental profiles. The results demonstrate that no packaging system simultaneously maximized product quality and environmental sustainability, highlighting the importance of integrating analytical performance with life-cycle assessment when developing innovative food packaging solutions.

## 1. Introduction

Apple (*Malus domestica* Borkh) is one of the most cultivated fruits worldwide. In 2023, global production reached 83.1 million metric tons, with China accounting for 56% of the total, followed by Europe (13%), Turkey (5.8%), and the United States (5.3%) [1,2]. At the European level, Italy ranks second with an estimated production of 2.2 million metric/tons in 2023 [1]. Although over 7500 apple cultivars are produced worldwide, only a few dominate the European market, including ‘Golden Delicious’ (24%), ‘Gala’ (9%), ‘Idared’ (7%), ‘Granny Smith’ (5%), and ‘Red Delicious’ (5%), globally representing about 59% of the total production [2].

One of the major factors contributing to the global apple market is the growing consumer awareness of the health-promoting benefits of food. From a nutritional perspective, apples are recognized as a valuable dietary source of antioxidants, primarily present as phenolics, including flavonoids and phenolic acids. Noteworthy, phenolic compounds mainly found in apples are chlorogenic acid, phloridzin, procyanidin B1 and B2, catechin, epicatechin, quercetin glycosides, and cyanidin-3-galactoside. Procyanidins represent the main phenolic class in both peel and flesh, followed by flavonols in the peel and hydroxycinnamic acids in the flesh. Literature research highlights that flavonoids and proanthocyanidins in apples offer a range of health benefits, including antioxidant, anticancer, antibacterial, anti-inflammatory, cardioprotective, immune-modulating, antimicrobial, antiproliferative, antiangiogenic, antihypertensive, as well as neuroprotective and anti-ageing effects [3]. The composition of phenolics in apples largely depends on the apple cultivar, agricultural practices, pedo-climatic conditions [4], and fruit maturity, with variations in distribution between peel and flesh observed across different cultivars [5,6].

Phenolic compounds play crucial roles in the genetically regulated ripening process of climacteric fruits, as they influence several physiological and biochemical pathways related to flesh softening, pigment synthesis, and the formation of volatile and aromatic compounds [7]. It follows that these secondary metabolites and their metabolic routes can impact the fruit’s shelf life and sensory qualities (i.e., browning, bitterness, and astringency), serving as substrates for enzymatic activities during post-harvest operations [3]. In this sense, minimally processed fruits have a short shelf life due to increased susceptibility to microbial spoilage, increased oxygen levels, ethylene production, and cell rupture that negatively affects the quality of cut apples. Polyphenol oxidase (PPO) stands out as the main enzyme involved in the browning process of apple flesh. PPO resides in plastids within plant cells, while phenolic compounds are stored in vacuoles [3]. However, when plant tissues incur damage, such as slicing or bruising during handling, the plastids and vacuoles rupture, promoting interactions between the phenolic compounds and PPO. In the presence of oxygen, PPO catalyzes the oxidation of phenols to form quinones, which then polymerize to produce brown, insoluble pigments such as melanin.

As cut and minimally processed apples are highly perishable, it is important to preserve their nutritional value and prolong shelf life, also exploiting innovative biodegradable materials supporting both quality and environmental compatibility. Innovative packaging is intended as natural-based films made up of biodegradable or edible polymers (such as corn starch, sugar cane, maize, etc.), supporting food quality and reducing the cost and the amount of traditional fossil-based packaging, such as plastic polymers [8,9], whose disposal strongly affects the environment, as 8 million tons [2,8] of plastic waste annually enter the ocean [8]. The relevance of studying fresh-cut apples is further supported by the growing market demand for ready-to-eat fruit products, which are washed, sliced, and packaged before consumption. Unlike whole apples, these minimally processed products lose part of their natural physical protection during processing and therefore are more susceptible to enzymatic browning, moisture loss, oxidative degradation, and microbial spoilage during refrigerated storage. Consequently, packaging plays a crucial role in maintaining quality attributes, extending shelf life, and reducing food waste throughout distribution and retail.

In this context, this study aimed to evaluate quality and sustainability assessment through the analysis of antioxidants, total phenolic content (TPC), and flavonoid content (TFC) using UV–Vis spectrophotometric assays. In addition, the phenolic profile of six compounds ((+)-catechin, caffeic acid, (−)-epicatechin, *p*-coumaric acid, rutin, and quercetin) was evaluated through HPLC-PDA in Golden Delicious apples obtained from conventional farming. The study evaluated traditional and innovative packaging types at 0, 14, and 21 days of storage. Innovative materials included corn starch, cassava, and eucalyptus-based film (Pack 2), polyethylene (PE) film (Pack 1), and polylactic acid (PLA)-based film (Pack 3) [10]. The environmental performance of both packaging categories was assessed using Life Cycle Assessment (LCA) methodology [11] with SimaPro v.9.5.5 software. A cradle-to-gate approach was applied to compare packaging materials and their environmental impacts.

## 2. Results and Discussion

### 2.1. Total Polyphenols, Flavonoids Content, and Antioxidant Activity by UV–Vis Spectroscopy

**Figure 1:**
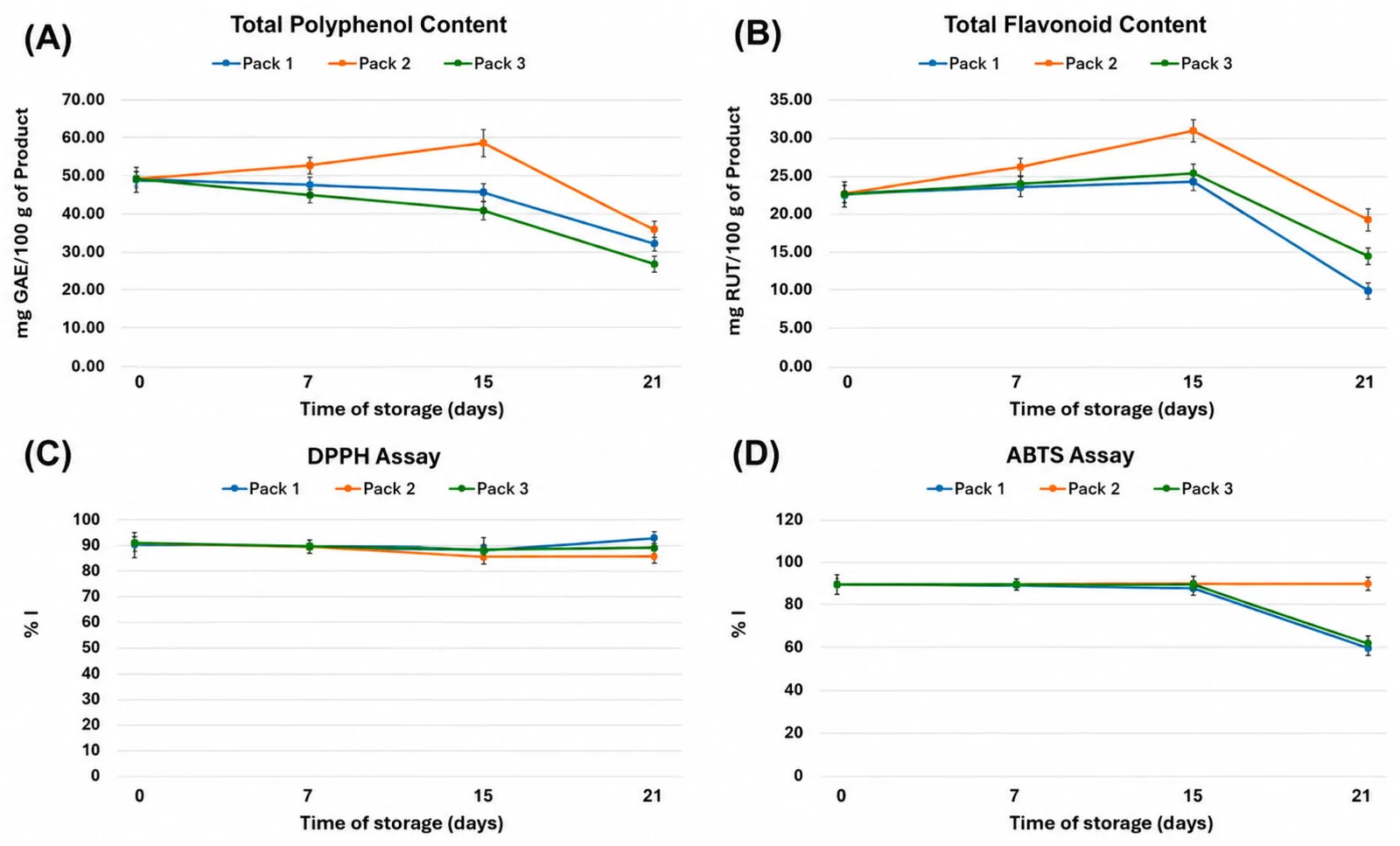
Total polyphenols content, total flavonoids content, and antioxidant activity by UV–Vis spectroscopy: (**A**) total phenolic content (TPC) expressed as mg GAE/100 g of products (±SD); (**B**) total flavonoid content (TFC) expressed as mg RE/100 g of products (±SD); (**C**) DPPH assay and (**D**) ABTS assay expressed as inhibition percentage (I%) in ready-to-eat apple samples during different storage times and packaging. Tested at 0 (t0), 14 (t14), and 21 days (t21). GAE: gallic acid; RE: routine, Pack 1 (PE), Pack 2 (corn starch, cassava, and eucalyptus bio-based film), and Pack 3 (polylactic acid (PLA) derived from corn starch).

To assess the total polyphenol content (TPC) in conventional Golden Delicious apples, the Folin–Ciocâlteu assay was used, comparing different packaging types (Pack 1, Pack 2, and Pack 3) and storage times (0, 14, and 21 days) (Figure 1).

The results highlighted a constant trend in TPC according to packaging type over time, except for a significant variation at t21. In fact, during the 21-day storage period, the TPC increased by an average of +36% compared to the whole fruit at t0. When considering the film composition used for storage, Pack 2 resulted in better-preserved phenolic content along the storage period at t14 and t21 (58.61 mg GAE/100 g of product; 36.4 mg GAE/100 g of product, respectively). Samples stored in Pack 2 showed an increase of approximately +30% in the content of total polyphenols and flavonoids (66.96 mg GAE/100 g of product; 36.58 mg RE/100 g of product, respectively) during storage at t21 (Figure 1). This increase may be linked to ethylene, a plant hormone that accelerates fruit ripening [12].

In addition, cutting and minimal processing may lead to a browning process in the fruit (including apples); in fact, the wound caused by cutting, cracking, or breaking apples produces a signal that migrates through the tissue and induces the synthesis of enzymes in a metabolic pathway responsible for the increased production of phenolic compounds and the resulting browning. Polyphenol oxidase (PPO) is the main enzyme responsible for the decolorization of sliced apple flesh by catalyzing the oxidation and reduction in natural phenolic substances into colourless quinones that subsequently polymerize to produce melanin-like coloured compounds responsible for browning [8]. To this purpose, research to improve the shelf life and quality of apples has also focused on the enzymatic inhibition of the phenylalanine-ammonia lyase (PAL), which is the rate-limiting enzyme of the phenylpropanoid pathway, generally induced by wounding [4]; in particular, an increase in PAL activity leads to an increase in phenolic compound content. Furthermore, it is worth mentioning that other factors, such as cultivation methods, apple varieties, and harvest seasons, can significantly influence the antioxidant content in apples [13].

Considering antioxidant activity, a consistent trend was observed among apple samples over the 21-day storage period, depending on the type of packaging used (Figure 1). However, at day 21 (t21), a 32% decrease in antioxidant content, as measured by the ABTS assay, was observed in apples stored in Pack 3 and Pack 1, compared to day 0. This reduction is likely due to the time-dependent oxidation of compounds and the differing gas permeability properties of the packaging materials [13]. As shown in Figure 1D, the DPPH radical scavenging activity was notably higher than ABTS activity, particularly in apples stored in Pack 2 (+15.44%) and Pack 3 (+11.61%). This suggests that hydrophobic antioxidant compounds in these apples may exhibit greater reactivity than their hydrophilic counterparts [14]. In this regard, the higher DPPH scavenging activity observed in Pack 2 and Pack 3 compared with ABTS activity should be evaluated in terms of the relative contribution of hydrophilic and less polar antioxidant compounds; in this sense, DPPH and ABTS assays are based on different radical species and may differ in reaction kinetics, accessibility of antioxidant molecules, and reaction mechanisms [9,11]. Therefore, the present data do not allow the observed differences to be attributed specifically to the presence of hydrophobic antioxidant compounds.

### 2.2. Polyphenol Quantification by HPLC-PDA

Table 1 shows the results obtained for the individual polyphenols in apple samples stored in various packaging materials (Pack 1, Pack 2, and Pack 3). Different polyphenols ((+)-catechin, (−)-epicatechin, *p*-coumaric acid, caffeic acid, rutin, quercetin) were analyzed at different storage times: at the time of sampling (t0), after 14 days (t14), and after 21 days (t21) of storage. Catechin (34.97 mg/100 g of product at t0) and epicatechin (31.05 mg/100 g of product) at t0 were the most commonly present in the samples analyzed. It is acknowledged that a few factors may influence the variability of polyphenol content. These include cultivar, cultivation techniques, and soil and climate conditions. Furthermore, in the presence of abiotic stress (e.g., UV radiation, drought, extreme temperatures) or biotic stress (e.g., pathogens, wounds), an increase in the activation of the phenylpropanoid biosynthetic pathway can be observed, increasing phenolic production [13,15].

Rutin (19.88 mg/100 g of product) and p-coumaric acid (13.87 mg/100 g of product) were also detected at t0, whereas caffeic acid was present at a lower concentration (0.23 mg/100 g of product). In apples, caffeic acid occurs predominantly in esterified forms, particularly as 5-caffeoylquinic acid (chlorogenic acid), which accounts for approximately 92.3% of the total caffeic acid derivatives [16,17]. Measured caffeic acid concentration may also be influenced by the acidic extraction conditions (methanol containing 1% HCl), which can promote partial hydrolysis of caffeoylquinic acids and release caffeic acid. Thus, the values obtained may reflect both free caffeic acid originally present in the samples and caffeic acid released during extraction. This aspect should be considered when interpreting changes in caffeic acid concentration during storage and among the different packaging conditions [18,19]. Chlorogenic acid represents an important storage form of caffeic acid in plant tissues and is localized in vacuoles and cell walls, where it contributes to protection against oxidative stress [18]. Free caffeic acid is also more susceptible to oxidation mediated by polyphenol oxidase.

The analysis revealed the presence of quercetin (2.37 mg/100 g of product) in low concentrations, as glycosylated forms such as quercetin-3-O-glucoside and quercetin-3-O-rutinoside (rutin) predominated. However, it should be noted that the quantification of these forms requires either acid or enzymatic hydrolysis [20]. Consequently, the measured concentration may be lower than the actual concentration. Indeed, previous studies showed that quercetin occurs almost exclusively in bound form (99.8%), while the free form accounts for less than 0.2% of the total content [21,22].

The study also investigated the variability of phenolic content during storage (t0, t14, and t21) of the fruit at refrigerated temperature, using different packaging made of various plastic materials (Pack 1, Pack 2, and Pack 3). During the storage period, it was determined that the concentration of polyphenols is influenced not only by the time of analysis but also by the type of material employed for packaging. Indeed, an increase in polyphenols is observed for Pack 2 between time t0 and t21 (+17.4%), while a reduction is evident for Pack 1 (−46.8%) and Pack 3 (−37.6%). This is presumably due to the differing gas exchange capacities (O_2_/CO_2_) and the varying light and oxygen permeability of the plastic materials used [23,24]. Catechin and epicatechin demonstrate the most significant variations over time (Table 1). Specifically, an increase in catechin concentrations is observed at day 21 in samples stored in Pack 2, while with Pack 3, an increase is observed at 14 days, followed by a decline at day 21. Referring to Pack 1, a peak in epicatechin is observed at day 14, with a subsequent more pronounced decline at day 21. The trends can be attributed to the depolymerisation processes of proanthocyanidins, which are caused by changes in the oxygen concentration inside the fruit [13,25]. *p*-Coumaric acid follows a constant degradation kinetics in all treatments, while rutin and quercetin remain at low concentrations and show no significant changes during storage.

### 2.3. Life Cycle Assessment of Packaging Materials

The packaging was also analyzed from an environmental perspective to determine the most sustainable choice for preserving fresh-cut apples. The results showed that the choice of packaging material affects the overall environmental impact of fresh-cut apple packaging.

Table 2 shows the 18 impact categories affected by the packaging under consideration. Polyethylene (PE), which is widely used in the food industry due to its favourable mechanical and barrier properties [26,27], was found to be the material with the worst performance in terms of global warming potential (5.23 × 10^−3^ kg CO_2_ eq) and fossil resource scarcity (2.58 × 10^−3^ kg oil eq), which is consistent with its petrochemical nature. However, the presence of well-established mechanical recycling chains facilitates its recovery, potentially reducing its environmental impact at the end of its life cycle.

**Figure 2:**
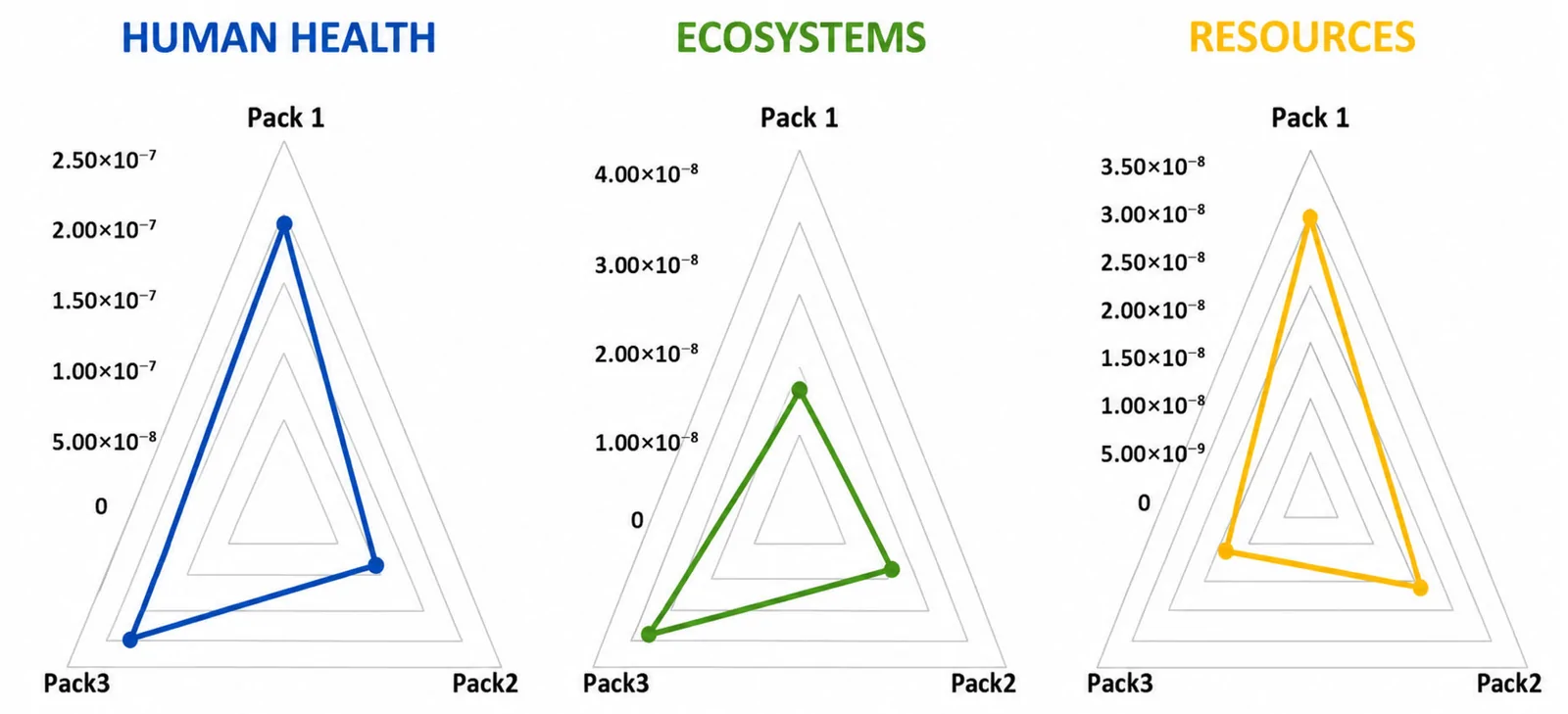
Normalized endpoint results for fresh-cut apples stored under three different packaging conditions.

Pack 3, a bioplastic derived from renewable resources, stands out for its ability to significantly reduce impacts associated with fossil fuels and climate-changing emissions. In this respect, it shows clear improvement compared to PE [27]. However, the analysis also reveals critical issues related to agricultural land use (7.59 × 10^−4^ m^2^ crop eq) and terrestrial ecotoxicity (5.91 × 10^−3^ kg 1,4-DCB). Also, the endpoint analysis (Figure 2) highlighted differences between the three packaging materials used, related to the material they are made of. About resource scarcity, Pack 1 has the greatest impact, followed by Pack 3, while Pack 2 performs best, with significantly reduced resource consumption. However, in terms of its impact on the ecosystem, particularly on the land use category, the situation is reversed: Pack 2 and Pack 3 demonstrate higher values than Pack 1, with Pack 2 demonstrating a slightly higher value than Pack 2. In this regard, it is worth mentioning that the values reported for biodegradable materials are not fully representative, as they do not account for the end-of-life stage of bioplastics, which usually involves their conversion into compost in industrial composting facilities. The production of compost and its subsequent application to agricultural soil as an amendment enables the return of carbon to the soil, thereby preventing carbon loss from the soil [8]. Finally, in terms of human health, Pack 2 has the greatest impact, surpassing both Pack 1 and Pack 3, which is the material with the lowest values in this category. The endpoint analysis confirmed that none of the alternatives considered is optimal in all impact categories. The data shows that no single alternative is ideal in every impact category, highlighting the necessity for an all-encompassing and multifaceted approach to evaluating the sustainability of materials.

A limitation of the present LCA is the adoption of a cradle-to-gate system boundary, which excludes the end-of-life stage of the packaging materials. This choice was made because reliable and comparable information on the actual end-of-life management of the different packaging systems under the conditions considered in this study was not available. Consequently, the present assessment does not capture potential environmental benefits or burdens associated with the biodegradability or compostability of the innovative packaging materials. The comparison should therefore be interpreted as a characterization of the environmental impacts associated with packaging production and use up to the defined system boundary, rather than as a complete assessment of the overall life cycle performance. Future studies should extend the system boundary to include end-of-life scenarios for all packaging materials under harmonized and realistic waste-management assumptions.

### 2.4. Chemometric Results

Chemometric analysis showed that storage time and packaging significantly influenced the phenolic composition and antioxidant activity of fresh-cut apples. When all seven experimental conditions, including the un-packaged t0 baseline, were considered, significant differences were observed for TPC (F6, 14 = 262.79, *p* < 0.001), TFC (F6, 14 = 291.58, *p* < 0.001), and ABTS activity (F6, 14 = 72.11, *p* < 0.001), whereas DPPH activity did not differ significantly among conditions (F6, 14 = 2.26, *p* = 0.098). To specifically assess the effects of storage time and packaging, a two-way ANOVA was performed on t14 and t21 samples. Storage time significantly affected TPC, TFC, and ABTS activity (*p* < 0.001), whereas DPPH activity was not significantly affected (F1, 12 = 1.01, *p* = 0.335). Packaging significantly affected TPC, TFC, and ABTS activity (*p* < 0.001), but not DPPH activity (F2, 12 = 0.75, *p* = 0.492). A significant storage time × packaging interaction was observed for TPC, TFC, and ABTS activity (*p* < 0.001), but not for DPPH activity (F2, 12 = 0.97, *p* = 0.407). These results indicate that the effects of packaging on phenolic content and ABTS activity depended on storage duration, while DPPH activity remained comparatively unaffected. Nevertheless, the higher TPC observed for Pack 2 did not correspond to the highest ABTS activity, indicating that total phenolic content alone does not fully explain antioxidant capacity. Although TPC and ABTS were positively correlated (r = 0.6885, *p* < 0.001), the relationship was not complete, suggesting that the qualitative composition of individual phenolic compounds may also influence the antioxidant response. This is supported by the significant correlations between ABTS and several individual phenolic compounds, including p-coumaric acid, caffeic acid, epicatechin, and catechin. However, in the absence of compound-specific antioxidant assays, this remains a possible explanation rather than a demonstrated causal relation.

For the HPLC data, each phenolic compound was analyzed separately using the same statistical approach. Across all seven conditions, significant differences were observed for catechin, epicatechin, caffeic acid, p-coumaric acid, rutin, and quercetin (*p* < 0.001). In the two-way ANOVA, storage time significantly affected all six compounds (*p* ≤ 0.0025), while packaging significantly affected epicatechin, caffeic acid, p-coumaric acid, rutin, and quercetin (*p* < 0.001), but not catechin (F2, 12 = 0.10, *p* = 0.906). Significant storage time × packaging interactions were observed for all compounds (*p* < 0.001), indicating that their changes during storage depended on the packaging material.

**Figure 3:**
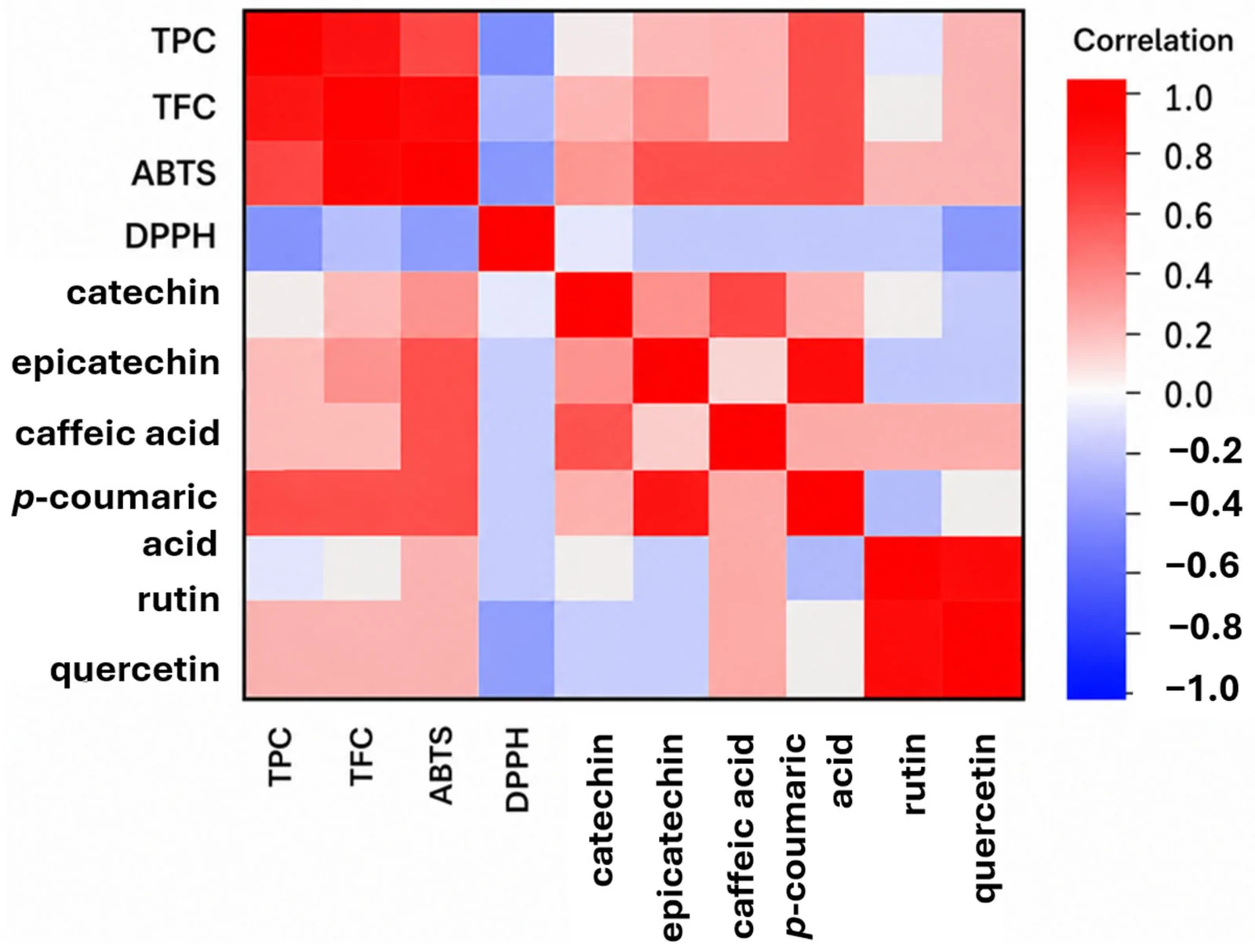
Heatmap showing the correlations between analytes (quercetin, rutin, p-coumaric acid, caffeic acid, catechin, and epicatechin) and spectrophotometric assays performed (TPC, TFC, ABTS, and DPPH).

Figure 3 shows the Pearson correlation heatmap obtained for the biochemical parameters analyzed during storage. Strong positive correlations were observed among the global spectrophotometric parameters, particularly between TPC and TFC (r = 0.8602, *p* < 0.001), TPC and ABTS activity (r = 0.6885, *p* < 0.001), and TFC and ABTS activity (r = 0.8100, *p* < 0.001). These relationships indicate that variations in total phenolic and flavonoid contents were associated with changes in ABTS antioxidant activity. Consistent with this pattern, ABTS activity was positively correlated with catechin (r = 0.5822, *p* = 0.0056), epicatechin (r = 0.6103, *p* < 0.001), caffeic acid (r = 0.6911, *p* < 0.001), and p-coumaric acid (r = 0.7221, *p* < 0.001), suggesting that several individual phenolic compounds may contribute to the observed antioxidant response. In contrast, the correlations between DPPH activity and TPC (r = −0.4164), TFC (r = −0.3877), and ABTS (r = −0.4554) were weaker, indicating that DPPH and ABTS may respond differently to changes in the phenolic profile. Among the individual phenolic compounds, quercetin and rutin showed a strong positive correlation (r = 0.8801, *p* < 0.001), while catechin and epicatechin showed a moderate positive correlation (r = 0.4919). Overall, the correlation analysis suggests that the antioxidant response cannot be attributed to a single phenolic compound but rather reflects the combined contribution of several components of the phenolic profile.

**Figure 4:**
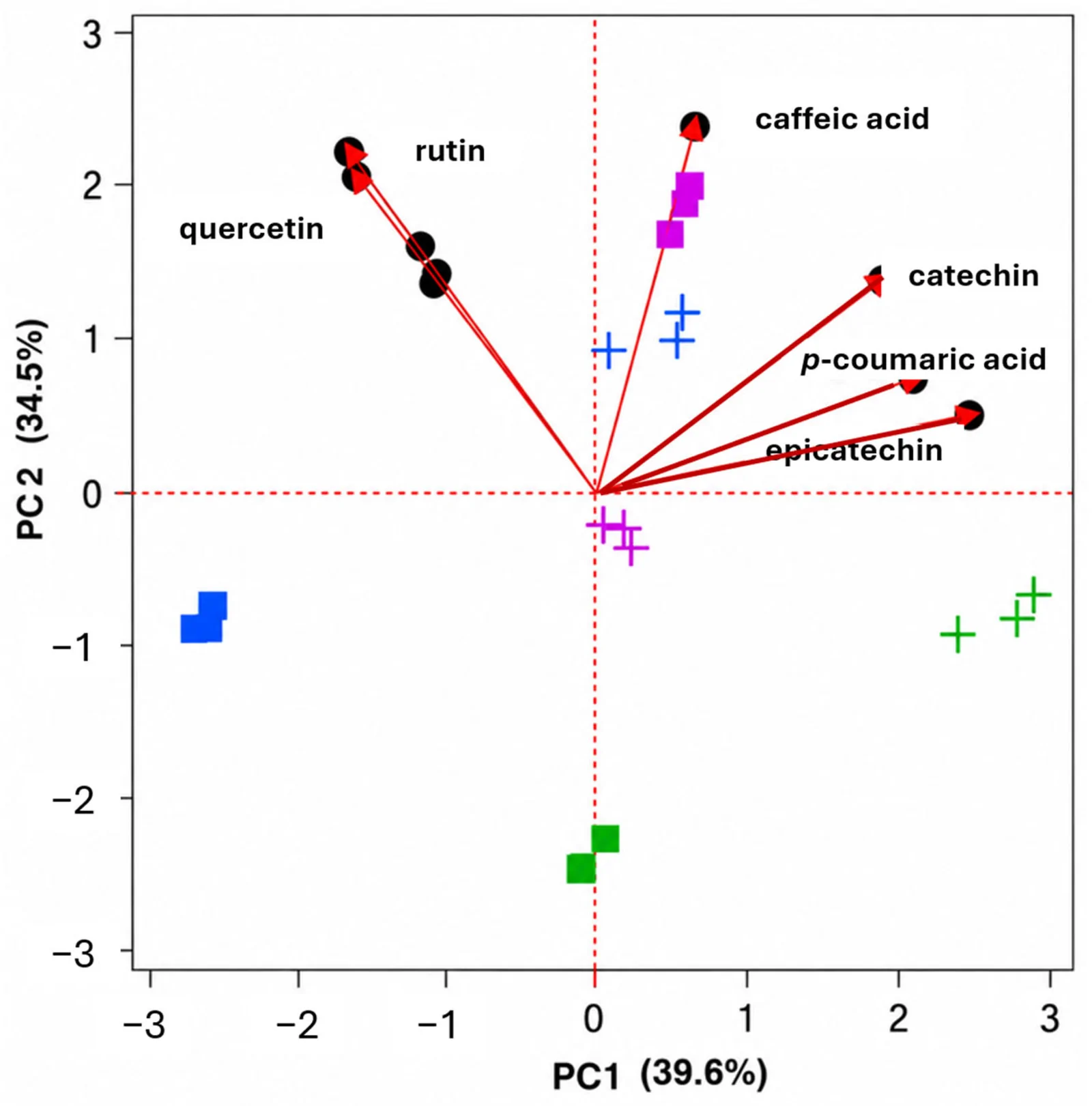
Biplot of the two principal components, accounting for 74.1% of the total variance. The symbols denote the sampling time (circle = t0, plus = t14, and square = t21), while the colours indicate the sample type (black = base sample, green = apples with Pack 1, pink = apples with Pack 2, and blue = apples with Pack 3).

Furthermore, high correlations are observed between catechins and epicatechin (r = 0.6911), indicating that these flavanols contribute significantly to antioxidant activity. Similarly, quercetin and rutin show a strong mutual correlation (r = 0.8801), indicative of a common biosynthetic pathway or accumulation. The weakness of some correlations, such as TPC-catechin, suggests that the polyphenolic profile depends on multiple polyphenolic compounds. Before principal component analysis (PCA), Pearson correlation analysis was performed to assess the linear relationships among the biochemical variables. PCA showed that the first two principal components, PC1 and PC2, explained 39.6% and 34.5% of the total variance, respectively, accounting for 74.1% of the cumulative variance. The biplot (Figure 4) shows that catechin, epicatechin, and p-coumaric acid contributed strongly to PC1, whereas PC2 was mainly associated with quercetin and rutin. Caffeic acid showed an intermediate contribution to both components. Overall, the multivariate analysis supports the presence of distinct changes in the phenolic profile during storage and indicates that packaging conditions may modulate the composition of individual bioactive compounds and the resulting antioxidant responses.

## 3. Materials and Methods

### 3.1. Chemicals

Folin–Ciocâlteu reagent, sodium carbonate (Na_2_CO_3_), 2,2-Diphenyl-1-picrylhydrazyl (DPPH), 2,2′-azino-bis (ABTS), sodium nitrite (NaNO_2_), aluminum chloride (AlCl_3_), methanol (CH_3_OH), ethanol (CH_3_CH_2_OH), double-distilled water (d-H_2_O), acetonitrile (CAN, >99% HPLC-grade), and acetic acid (HPLC-grade), as well as polyphenol standards (+)-catechin (≥98% HPLC purity), caffeic acid (≥95% HPLC purity), (−)-epicatechin (≥95% HPLC purity), *p*-coumaric acid (≥98% HPLC purity), rutin (≥98% HPLC purity), and quercetin (≥98% HPLC purity) were purchased from Sigma-Aldrich (St. Louis, MO, USA).

### 3.2. Sample Preparation

The Golden Delicious (*Malus domestica* Borkh.) apples were provided by Consorzio Melinda S.c.a. (Cles (TN), Val di Non, Italy). The apples were collected in May 2023 and transported to the analysis laboratory within six hours under refrigerated conditions (4 ± 2 °C). Upon arrival, the apples were weighed and cut, and the core and any visible defects were removed. Different aliquots (150 ± 4 g) were subsequently prepared and packaged using three different materials. Commercial polyethylene (PE) was used as the reference packaging film (Pack 1), while two innovative packaging materials were investigated: bio-based film from corn starch, cassava, and eucalyptus (Pack 2), and a polylactic acid (PLA) biofilm derived from corn starch (Pack 3).

Fresh-cut apples before packaging were analyzed at the initial time point (t0), which was considered the baseline condition. The packaged samples were then stored at 5 °C for 21 days, and analyzed after 14 days (t14) and 21 days (t21). At each post-packaging sampling time, samples from all three packaging systems were collected. Thus, the experimental design comprised seven sampling conditions in total: one baseline condition at t0 and three packaging conditions at each of the two subsequent storage times (t14 and t21).

The samples were subsequently lyophilized (Lyovapor™ L-200, Buchi, Milan, Italy) at 0.200 mbar for 12 h before extraction, as described in Section 2.3 and Section 2.4 for spectrophotometric and chromatographic analyses, respectively. Each experimental condition was analyzed in triplicate.

Following lyophilization, the dry mass was recorded and used to determine the dry matter fraction and the corresponding conversion factor from dry weight to fresh weight. The average moisture content of the analyzed samples was 90.65 ± 5.78%. For each sample, the moisture content was calculated as in Equation (1):

(1) $$\mathrm{Moisture}(\%)=\;\frac{m\;fresh-m\;dry}{m\;fresh}\;\times \;100$$

where m_fresh_ and m_dry_ represent the sample mass before and after lyophilization, respectively. Analytical results obtained on a dry-weight basis were converted to a fresh-weight basis using the sample-specific dry matter fraction (Equation (2)):

(2) $$X\;fw=X\;dry\;\times \frac{m\;dry}{m\;fresh}$$

The resulting values were expressed per 100 g of fresh sample and determined individually for each sample from its corresponding fresh and lyophilized masses.

### 3.3. Polyphenol, Flavonoid, and Antioxidant Activity Assays

The extraction of antioxidants from Golden Delicious apple samples was conducted following the method of Di Matteo et al. (2021) [10], with slight modifications. Briefly, 0.25 g (±0.01 g) of the sample was added to 10 mL of an ethanol aqueous solution (70:30, *v*/*v*), followed by sonication at 30 °C for 35 min (Bandelin Sonorex RK100H, Berlin, Germany). The mixture was centrifuged at 3000 rpm for 10 min at 25 °C (NEYA 10R, Exacta Optech, Milan, Italy). This process was repeated twice, with a final volume of 20 mL.

#### 3.3.1. Total Polyphenol Content

The total polyphenol content (TPC) was measured by mixing 0.5 mL of the hydroalcoholic extract with 0.25 mL of Folin–Ciocâlteu reagent in a 10 mL amber volumetric flask, following the method of Di Matteo et al. (2021) [10]. After 3 min, 0.5 mL of an aqueous sodium carbonate solution (7.5% *w*/*v*) was added, and the flask was kept in the dark for 30 min. The solution was then filled up to volume with distilled water (d-H_2_O). The absorbance was read at λ = 750 nm. Results were expressed as milligrams of gallic acid equivalents per 100 g of fresh apple weight (mg GAE/100 g of products), based on a calibration curve ranging from 10 to 100 mg/L (R^2^ = 0.9998), with the extraction solvent used as the blank.

#### 3.3.2. Total Flavonoid Content

The total flavonoid content (TFC) was determined using the method of Abdel-Naeem et al. (2021) [12], with slight modifications. In a 5 mL volumetric flask, 0.5 mL of extract was mixed with 2 mL of distilled water and 150 µL of sodium nitrite (5% *w*/*v*). After stirring and incubating the solution in the dark for 5 min, 150 µL of aluminum chloride (10% *w*/*v*) was added, followed by an additional 5 min incubation in the dark. Then, 2 mL of sodium hydroxide (1 M) was added, and the solution was left in the dark for 15 min. The final volume was adjusted to 5 mL. Absorbance was measured at 510 nm. TFC results were expressed as milligrams of rutin equivalents per 100 g of fresh apple (mg RE/100 g of product).

#### 3.3.3. Antioxidant Activity by ABTS and DPPH Assays

The antioxidant activity was assessed using two different assays: ABTS and DPPH. The ABTS radical scavenging activity was determined by measuring the reduction in absorbance at 734 nm. In this assay, 0.4 mL of the hydroalcoholic extract was mixed with 3.6 mL of ABTS reagent and kept in the dark for 15 min before reading the absorbance using a UV–Vis spectrophotometer.

For the DPPH assay, 1 mL of hydroalcoholic extract was combined with 1.5 mL of DPPH solution (2.5 ng/mL) and incubated in the dark for 30 min at room temperature. The absorbance was measured at 517 nm against methanol. The antioxidant activity in both assays was calculated based on the inhibition rate (I%) of the radical cation, using the following equation (Equation 3):

I% = (A_0_ − A_1_)/A_0_ (3)

where A_0_ is the absorbance of the control (blank), and A_1_ is the absorbance of the extract’s DPPH or ABTS radical activity.

### 3.4. HPLC-PDA Analysis of Phenolic Compounds

#### 3.4.1. Phenolic Compounds Extraction

Single phenolic compound extraction followed the method of Tarola et al. (2019) [28] with minor modifications. Briefly, 0.25 g (±0.01 g) of lyophilized apple sample was added to 5 mL of methanol containing 1% hydrochloric acid (MeOH/1% HCl), homogenized in an ultrasonic bath at 25 °C for 10 min, and centrifuged at 3000 rpm for 10 min at 25 °C. This process was repeated twice, with a final volume of 10 mL. The extract was then evaporated under N_2_ flow to a final volume of 5 mL and filtered through a 0.45 µm filter (Whatman^®^ Puradisc filters, Sigma-Aldrich, Milan, Italy), before the chromatographic analysis.

#### 3.4.2. Identification and Quantification

The HPLC instrument was a 40-AVP Shimadzu (Shimadzu, Tokyo, Japan) with a photodiode array detector (PDA) SPD-M40 and a binary pump. Chromatographic separation was performed on a Supelcosil RP LC-18 (15 cm×4.6 mm, 3 µm) column (Sigma-Aldrich, St. Louis, MO, USA) at room temperature.

The elution method was performed according to Alberti et al. (2014) with some modifications [29]. The mobile phase was composed of solvent A (2.5% acetic acid in ultrapure water, *v*/*v*) and solvent B (acetonitrile). The following gradient was used: 3–14% B (0–5 min), 14–16% B (5–15 min), 16–36% B (15–33 min), followed by isocratic elution at 100% B (5 min) and then column reconditioning at 3% B for 10 min. The flow rate was 1.0 mL/min. A qualitative and quantitative analysis of phenolic compounds was performed by acquiring and comparing compound spectra and retention times with those of standards. The chromatograms were acquired at 280 nm (flavan-3-ols), 320 nm (hydroxycinnamic acids), and 350–370 nm (flavonols). Quantification was performed using standard calibration curves as illustrated in Table 3.

### 3.5. Life Cycle Assessment of Packaging Films

To provide better packaging options in terms of both product preservation and environmental impact, a comparative Life Cycle Assessment (LCA) [11] was conducted on the packaging examined in the study. This analysis was performed using SimaPro 9.5.5 software. A single-use bag (155 mm × 160 mm) was chosen as the functional unit (FU) for the three different types of packaging (Pack 1, Pack 2, and Pack 3), which were characterized by different plastic materials. The three types of packaging were analyzed according to their composition and specific weight (Table 4).

The different film thicknesses were accounted for in the LCA through the actual weight of each packaging unit. The material amount was calculated for each bag and related to the defined functional unit, so that differences in thickness and resulting material mass were directly incorporated into the environmental assessment.

The ReCiPe 2016 Endpoint (H) V1.05 method was used for the impact calculations, thus considering the following impact categories: global warming, human health (GW, HH); stratospheric ozone depletion (SOP); ionizing radiation, (IR); ozone formation, human health (OF, HH); fine particulate matter formation (FPMF); human carcinogenic toxicity (HCT); human non-carcinogenic toxicity (HCNT); water consumption, human health (WC, HH); global warming, terrestrial ecosystems (GW, TE); global warming, freshwater ecosystems (GW, FE); ozone formation, terrestrial ecosystems (OF, TE); terrestrial acidification (TA); freshwater eutrophication (FE); marine eutrophication (ME); terrestrial ecotoxicity (TE); freshwater ecotoxicity (FET); marine ecotoxicity (MET); water consumption, terrestrial ecosystem (WC, TE); water consumption, aquatic ecosystems (WC, AE); land use (LU); mineral resource scarcity (MRS); fossil resource scarcity (FRS). These categories are considered endpoint indicators showing the environmental impacts on three macro-categories: human health (HH), ecosystems (Es), and resources (Rs).

### 3.6. Statistical Analysis

All data are reported as mean ± standard deviation (dev. std.) of three replicates. The distributions of each variable were evaluated using the Shapiro–Wilk and Levene tests. Because the t0 samples represented the un-packaged baseline condition, whereas the three packaging treatments were evaluated at t14 and t21, the statistical analysis was structured according to the experimental design. A one-way ANOVA was first performed considering the seven experimental conditions (t0 and the three packaging treatments at t14 and t21) as a single factor, followed by Tukey’s post hoc test to identify significantly different pairs of conditions. To specifically evaluate the effects of storage time and packaging during storage, a two-way ANOVA was then performed on the t14 and t21 samples, incorporating the factors “Time” and “Packaging”, as well as their interaction. This analysis was performed separately for each response variable and for each phenolic compound determined by HPLC. The experimental effect was considered significant at *p* < 0.05. Post hoc comparisons were performed on least-squares means using Tukey’s method. A Pearson correlation analysis was performed between the biochemical parameters using the “Multivariate” function of JMP^®^ Pro 17 software. The correlation coefficients (r) and respective *p*-values were used to evaluate the strength and significance of the linear relationships between the variables. PCA was conducted on the biochemical parameters using the “Principal Components” function of JMP^®^ Pro 17 software (SAS Institute Inc., Cary, NC, USA) to reduce the dimensionality of the data and identify patterns associated with the treatments.

## 4. Conclusions and Future Perspectives

This study investigated the impact of various packaging materials on the preservation of bioactive compounds in ready-to-eat Golden Delicious apples. After 21 days of storage, significant differences emerged in the stability of phenolic compounds and antioxidant activity among the three tested packaging films (Pack 1, Pack 2, and Pack 3). These variations were closely linked to fruit physiology: cutting and handling activate metabolic pathways involved in polyphenol synthesis (i.e., PAL activity), and the permeability of the packaging to gases modulates the accumulation or degradation of these compounds. In detail, key findings for the tested packaging films included:

Pack 1 resulted in the lowest preservation of polyphenols (−31% at day 21), but its environmental profile was more favourable in several impact categories, primarily due to its relatively efficient fossil-based production with lower land and water use.

Pack 2 showed an increase in polyphenols between time t0 and t21 (+17.4%), likely driven by altered gas exchange that stimulated ripening or stress-related responses. Correlations between antioxidant activity and compound-specific profiles, supported by PCA, further indicate that the qualitative composition of phenolics influences antioxidant capacity more than their total concentration.

Pack 3 denoted a reduction of −37.6% in total polyphenol content after 21 days of storage, with better performance in maintaining flavonoid content over time. However, despite its biobased origin, which reduces dependence on fossil resources, PLA was associated with a decline in antioxidant activity during storage. Additionally, the LCA study highlighted critical environmental burdens related to land and water use; its end-of-life management remains constrained by the limited availability of industrial composting facilities.

Moreover, it is important to underline that end-of-life benefits (e.g., compostability) do not outweigh the agricultural burdens in this dataset. In this regard, the possibility of composting bioplastics in soil amendments could restore carbon stocks, thus effectively mitigating soil carbon losses.

Overall, the combined quality and environmental assessment show that no single packaging material can be considered optimal. Instead, the design of packaging for fresh-cut produce should strike a balance that preserves nutritional quality while minimizing life-cycle environmental impacts and aligning with existing waste management and recycling infrastructure.

## Figures and Tables

**Table 1:** Concentration (mg/100 g product) of the six free polyphenols in apple samples stored at room temperature in different packaging materials (PLA, cassava, eucalyptus, corn starch-based film, and PE-based film). Values are expressed as the mean value of the three replicates (±standard deviation). The letters in superscript indicate the homogeneous groups identified by Tukey’s test (*p* < 0.05). Means sharing the same letter do not differ significantly from each other.

Phenolic Compounds	t_0_	t_14_			t_21_		
		Pack 1	Pack 2	Pack 3	Pack 1	Pack 2	Pack 3
(+)-catechin	34.97 ± 0.52 ^e^	46.65 ± 1.57 ^f^	30.65 ± 3.50 ^b^	57.33 ± 1.33 ^i^	36.27 ± 0.69 ^f^	51.36 ± 1.21 ^d^	24.82 ± 0.1.79 ^a^
(−)-epicatechin	31.05 ± 0.81 ^d^	52.68 ± 2.92 ^b^	32.24 ± 1.17 ^g^	32.05 ± 0.95 ^f^	23.93 ± 0.63 ^b^	35.49 ± 0.50 ^c^	23.4 ± 1.63 ^a^
*p*-coumaric acid	13.87 ± 0.74 ^d^	20.11 ± 0.68 ^b^	18.61 ± 0.48 ^h^	10.13 ± 0.77 ^c^	8.44 ± 0.005 ^c^	15.34 ± 0.37 ^f^	6.38 ± 0.65 ^a^
caffeic acid	0.23 ± 0.01 ^f^	0.17 ± 0.003 ^c^	0.20 ± 0.006 ^d^	0.25 ± 0.005 ^h^	0.18 ± 0.46 ^b^	0.27 ± 0.004 ^e^	0.14 ± 0.007 ^a^
rutin	19.88 ± 0.51 ^i^	7.26 ± 0.35 ^a^	9.78 ± 0.36 ^c^	15.34 ± 0.28 ^d^	0.67 ± 0.21 ^a^	15.81 ± 0.48 ^h^	17.65 ± 0.23 ^f^
quercetin	2.32 ± 0.06 ^i^	0.67 ± 0.02 ^a^	1.71 ± 0.02 ^d^	1.18 ± 0.05 ^c^	0.19 ± 0.008 ^a^	1.84 ± 0.11 ^h^	1.93 ± 0.09 ^f^
**Total**	**102.32**	**127.54**	**93.18**	**116.28**	**69.68**	**120.11**	**74.32**

**Table 2:** Environmental impacts of the three different packaging films (PLA, cassava, eucalyptus, corn starch-based film, and PE-based film) used to preserve fresh-cut apple samples.

Impact Category	Unit	Pack 1	Pack 2	Pack 3
**Global warming**	kg CO_2_ eq	5.23 × 10^−3^	4.46 × 10^−3^	3.37 × 10^−3^
**Stratospheric ozone depletion**	kg CFC_11_ eq	4.95 × 10^−10^	8.23 × 10^−9^	3.15 × 10^−9^
**Ionizing radiation**	kBq Co-60 eq	3.39 × 10^−4^	2.19 × 10^−4^	2.29 × 10^−4^
**Ozone formation, human health**	kg NO_x_ eq	9.68 × 10^−6^	1.01 × 10^−5^	7.05 × 10^−6^
**Fine particulate matter formation**	kg PM_2.5_ eq	1.54 × 10^−6^	2.63 × 10^−6^	9.23 × 10^−7^
**Ozone formation, terrestrial ecosystems**	kg NO_x_ eq	1.06 × 10^−5^	1.08 × 10^−5^	7.77 × 10^−6^
**Terrestrial acidification**	kg SO_2_ eq	1.16 × 10^−5^	1.61 × 10^−5^	1.20 × 10^−5^
**Freshwater eutrophication**	kg P eq	1.20 × 10^−6^	1.74 × 10^−6^	1.07 × 10^−6^
**Marine eutrophication**	kg N eq	1.28 × 10^−7^	1.69 × 10^−6^	6.35 × 10^−7^
**Terrestrial ecotoxicity**	kg 1,4-DCB	3.36 × 10^−3^	5.91 × 10^−3^	5.43 × 10^−3^
**Freshwater ecotoxicity**	kg 1,4-DCB	9.87 × 10^−5^	1.72 × 10^−4^	1.42 × 10^−4^
**Marine ecotoxicity**	kg 1,4-DCB	2.94 × 10^−5^	4.77 × 10^−5^	4.06 × 10^−5^
**Human carcinogenic toxicity**	kg 1,4-DCB	1.34 × 10^−6^	1.26 × 10^−6^	1.15 × 10^−6^
**Human non-carcinogenic toxicity**	kg 1,4-DCB	5.54 × 10^−5^	9.71 × 10^−5^	8.37 × 10^−5^
**Land use**	m^2^a crop eq	1.15 × 10^−4^	7.59 × 10^−4^	3.52 × 10^−4^
**Mineral resource scarcity**	kg Cu eq	6.15 × 10^−6^	9.94 × 10^−6^	9.21 × 10^−6^
**Fossil resource scarcity**	kg oil eq	2.58 × 10^−3^	9.90 × 10^−4^	1.46 × 10^−3^
**Water consumption**	m^3^	3.80 × 10^−5^	1.68 × 10^−4^	7.10 × 10^−5^

**Table 3:** Chromatographic parameters of phenolic compounds analyzed by HPLC-PDA. Mean value (*n* = 3).

Phenolic Compound	Retention Time (min) ±SD	UV Bands (nm)	Linear Range (mg/L)	Regression Equation	R^2^	%RSD	LOD (mg/L)	LOQ (mg/L)
**(+)-Catechin**	8.15 ± 0.04	280	5–100	y = 574,044x − 10^7^	0.9992	0.51	0.07	0.26
**Caffeic acid**	10.02 ± 0.04	320	0.3–20	y = 162,518x − 6240.5	0.9976	1.02	0.03	0.09
**(−)-Epicatechin**	11.55 ± 0.03	280	5–100	y = 7028.9x + 63,216	0.9987	1.14	0.06	0.22
***p*-Coumaric acid**	14.42 ± 0.06	320	5–30	y = 142,093x – 6828.4	0.9975	1.19	0.03	0.13
**Rutin**	18.53 ± 0.02	350	4–20	y = 14,939x + 41,122	0.9992	0.71	0.26	0.83
**Quercetin**	27.88 ± 0.03	370	5–100	y = 40,996x − 23,123	0.9986	1.43	0.06	0.12

**Table 4:** Composition of commercial films, reported on the package label, and their main characteristics [10].

Packaging Film	Composition	Dimension (mm)	OTR (cm^3^ × pm^−2^ × day^−1^)	CO_2_TR (cm^3^ × m^−2^ × day^−1^)	Single-Sachet Weight (g)
**Pack 1**	High-density polyethylene (PE)	155 × 160 mm	5396 ± 33.7	26,240 ± 750.4	1.43
**Pack 2**	Corn starch, cassava, and eucalyptus	155 × 160 mm	4.30 ± 0.20	13.42 ± 0.71	1.51
**Pack 3**	Polylactic acid from corn starch (PLA)	155 × 160 mm	646.41 ± 32.33	2162.51 ± 110.13	1.22

CO_2_TR: CO_2_ transmission rate; OTR: O_2_ transmission rate.

## Data Availability

The data that support the findings of this study are available on request.
